# Monitoring of Fe(II) Spin Transition in Cu(II)-Doped Spin-Crossover Nanoparticles

**DOI:** 10.3390/molecules30061258

**Published:** 2025-03-11

**Authors:** Alexander Charitos, Vassilis Tangoulis, John Parthenios, Ondrej Malina, Radim Mach, Nikolaos Ioannidis, Nikolia Lalioti

**Affiliations:** 1 Laboratory of Inorganic Chemistry, Department of Chemistry, University of Patras, 26504 Patras, Greece; charitos.al97@gmail.com; 2 Institute of Chemical Engineering Sciences (ICE-HT), Foundation for Research and Technology-Hellas (FORTH), 26504 Patras, Greece; jparthen@iceht.forth.gr; 3 Regional Centre of Advanced Technologies and Materials, Czech Advanced Technology and Research Institute (CATRIN), Palacký University Olomouc, Šlechtitelů 27, 78371 Olomouc, Czech Republic; ondrej.malina@upol.cz (O.M.); radim.mach@upol.cz (R.M.); 4 Institute of Nanoscience and Nanotechnology, NCSR “Demokritos”, 15310 Athens, Greece; n.ioannidis@inn.demokritos.gr

**Keywords:** Fe-triazole, spin crossover, EPR, Raman, thermal hysteresis

## Abstract

Experimental protocols based on Electron Paramagnetic Resonance (EPR) and Raman spectroscopy are presented for the investigation of the Fe(II) spin transition in Cu(II)-doped 1-D spin-crossover (SCO) nanoparticles of the type [Fe_1−x_Cu_x_(NH_2_trz)_3_]Br_2_ where x = 0.03 and 0.06 and NH_2_trz = 4-amino-1, 2, 4-triazole. The resulting nanoparticles were characterized using Transmission Electron Microscopy (TEM), Infrared (IR) spectroscopy, and powder X-ray diffraction (p-XRD). Magnetic susceptibility measurements revealed a dependence on the scan rate, with critical temperatures and hysteresis widths varying accordingly. EPR spectroscopy provided insights into the doped nanoparticles’ structural changes and spin-state transitions. The Cu(II) dopants exhibited significant g-factor anisotropy and hyperfine structure, indicative of a distorted octahedral coordination. The EPR spectra indicated that the spin transition occurs in domains populated by ions of the same spin state. Cu(II) ions show different spectral characteristics depending on whether they are in high-spin or low-spin domains of Fe(II). Changes in Raman bands induced by laser power reveal structural and electronic rearrangements during the LS to HS transition. The findings provide insights into metal–ligand interactions and the molecular mechanisms underlying the SCO process.

## 1. Introduction

The EPR (Electron Paramagnetic Resonance) spectroscopy [1,2,3,4,5] is an essential analytical technique widely used for the investigation of spin-crossover (SCO) phenomena, with a particular focus on iron(II) complexes [6]. This technique is compelling as it allows researchers to probe the transitions between high-spin (HS) and low-spin (LS) states of SCO iron(II) complexes. Spin-state transitions can be effectively monitored by incorporating small amounts of EPR-active metal ions, such as manganese (II) and copper (II), into these complexes. The dopants are usually added in concentrations not exceeding 3–5% to preserve the inherent SCO characteristics of the iron(II) complex. This approach offers valuable insights into spin-state changes by analyzing the EPR spectra of the dopant ions [6].

One of the pivotal functions of EPR spectroscopy in studying SCO is its ability to provide insights into structural changes occurring within the host complex. The specific properties of Cu(II) and Mn(II) EPR spectra, particularly the anisotropy and the zero-field splitting observed in Cu(II) and Mn(II) ions, respectively, are remarkably sensitive indicators of the symmetry and local arrangement of the lattice sites that the dopants occupy [7,8,9,10,11,12,13]. Besides probing structural changes, EPR spectroscopy is integral in studying allosteric switching phenomena in materials where multiple metal sites experience SCO simultaneously. A compelling example is found in the compound [Fe(1-bpp)_2_]_z_[Co(terpy)_2_]_1−z_[BF_4_]_2_, where EPR specifically investigates the LS state of the [Co(terpy)_2_]^2+^ sites present [14]. EPR data from these studies indicate that LS dopants’ population mirrors the host lattice’s spin state during the Light-Induced Excited Spin-State Trapping (LIESST) experiment. This finding suggests that allosteric spin-state switching occurs among the iron and cobalt sites under such experimental conditions. This offers significant insights into the cooperative interactions among different metal sites within the material.

The EPR investigation of the Fe/triazole SCO system on a microscale has been investigated thoroughly by the pioneering work of Kahn et al. [15,16,17,18]. O. Kahn focused primarily on the behavior of Cu(II) and Mn(II) ions as paramagnetic probes within these systems. For the case of [Fe(NH_2_trz)_3_](NO_3_)_2_ [17,18] and at low temperatures, specifically at 113 K, the EPR spectrum for Cu(II) demonstrates significant g-factor anisotropy and a clear hyperfine structure, indicative of the ion occupying a distorted octahedral coordination bearing a Jahn–Teller effect. The average g-values and hyperfine coupling constants (A-values) observed at 113 K and 320 K show subtle yet noteworthy variations, suggesting the significant impact of motional averaging on distortions due to the Jahn–Teller effect experienced by the dopant Cu(II) ions. While in the case of a copper-doped nitrogen-containing 2D SCO Fe(II) system of the type [Fe(NCS)_2_(btr)_2_]H_2_O (btr = 4,4′-bi-1,2,4-triazole) the EPR spectra for Cu(II) exhibit a superhyperfine structure [19] associated with the ^14^N isotope, the EPR spectra of the Cu-doped [Fe(NH_2_trz)_3_](NO_3_)_2_ show no similar features. Nonetheless, certain degrees of inhomogeneous broadening are apparent, which might be attributed to unresolvable superhyperfine splitting. When the [Fe(NH_2_trz)_3_](NO_3_)_2_ system populates the HS state, the EPR spectra for the paramagnetic dopant ions of Cu(II) and Mn(II) become notably broadened due to strong interactions with paramagnetic Fe(II) ions. This broadening is further accompanied by the rapid spin–lattice relaxation of Fe(II) centers, which dynamically averages out the spin–spin interactions, impacting the apparent linewidths.

According to O. Kahn, the linewidths are influenced by a combination of dipole–dipole broadening and exchange interactions between paramagnetic dopant ions and Fe^2+^ centers [16,17,18]. The role of the rapid spin–lattice relaxation of the Fe(II) centers on linewidths is acknowledged through cross-relaxation mechanisms. In the case of [Fe(NH_2_trz)_3_](NO_3_)_2_, two adjacent Fe(II) ions are connected by amino–triazole ligands in a triply bridged manner. Conversely, in [Fe(Htrz)_2_(trz)]BF_4_, one of the three triazole bridges undergoes spontaneous deprotonation, resulting in a more significant distortion of the host sites. During the spin transition, the EPR spectra of Cu(II) and Mn(II) in Fe(NH_2_trz)_3_](NO_3_)_2_ demonstrate a gradual decrease in amplitude rather than exhibiting line broadening, which suggests the population of both LS/HS Fe(II) domains. This behavior contrasts with Fe(Htrz)_2_(trz), where the EPR spectra display distinct broadening characteristics influenced by structural differences and a higher spin transition temperature [20].

Inspired by the EPR study mentioned before, we decided to investigate the Fe(II) spin transition in Cu(II)-doped 1-D SCO NPs of the type [Fe_1−x_Cu_x_(NH_2_trz)_3_]Br_2_. Our group has established experimental protocols based on a reverse micellar approach for synthesizing [Fe(NH_2_trz)_3_]Br_2_ NPs and graphene-oxide-based hybrids of this family [21,22]. Our study has demonstrated that the variable ω_0_, which indicates the molar ratio of water to surfactant, alongside the duration of the reaction, is pivotal in shaping the morphology and properties of the thermal hysteresis. Remarkably, a two-step hysteresis was identified for the first time when employing a magnetic sweep rate of 1 K/min, specifically at ω_0_ = 10 and a reaction time of 20 h, and the resulting NPs will be hereinafter referred to as **Fe1**. This two-step hysteresis transitioned to a single-step hysteresis when the reaction time was 48 h, and the resulting NPs will be hereinafter referred to as **Fe2**. Elevating the magnetic sweep rate to 10 K/min resulted in detecting a single-step hysteresis characterized by critical temperatures of T_1/2_↑ = 330–335 K and T_1/2_↓ = 290–300 K [21].

In the present work, EPR spectroscopy was used to investigate the SCO phenomenon in Cu(II)-doped 1-D SCO NPs of the type [Fe_1−x_Cu_x_(NH_2_trz)_3_]Br_2_. TEM and PXRD measurements determined the morphology and dimensions of the SCO NPs. Thermal hysteretic behavior was investigated using sweep-rate-dependent magnetic measurements and differential scanning calorimetry. Temperature-dependent EPR measurements were carried out to monitor the origin and evolution of the SCO phenomenon. A micro-Raman study was conducted to monitor the SCO phenomenon following a power-dependent experimental protocol.

## 2. Results and Discussion

### 2.1. General Synthetic Aspects

In this study, we present the synthesis using a reverse micellar approach of the 1-D SCO Coordination Polymer (CP) [Fe(NH_2_trz)_3_]Br_2_, doped with 3% and 6% Cu(II) ions. This method involved using a non-ionic surfactant TX100, a n-hexanol as a co-surfactant, and a cyclohexane as the organic solvent with synthetic parameters ω_0_ = 10 and a reaction time of 24 h. Elemental and EDS analysis (Tables S1 and S2, Figure S1) confirmed the general formulas: [Fe_0.97_Cu_0.03_(NH_2_trz)_3_]Br_2_⋅2H_2_O⋅0.02TX100 (**Cu3**) and [Fe_0.94_Cu_0.06_(NH_2_trz)_3_]Br_2_⋅ 2H_2_O⋅0.02TX100 (**Cu6**).

**Figure 1:**
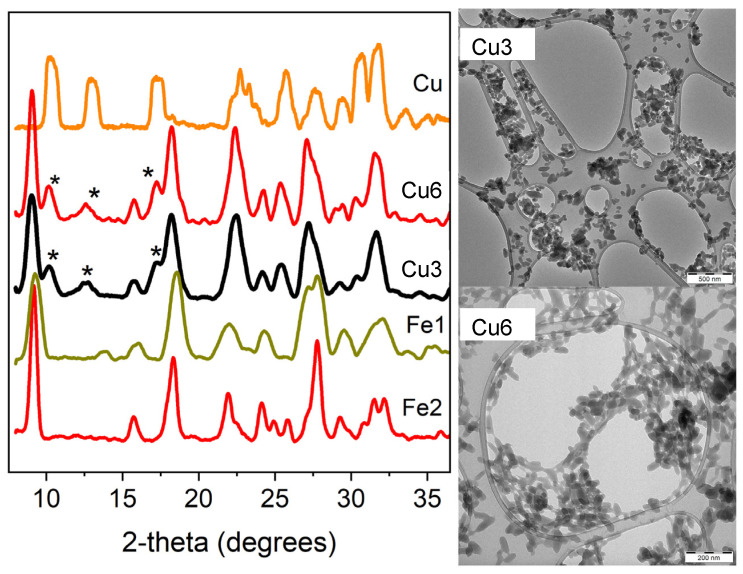
(**left**) p-XRD diffractograms of pristine **Cu, Fe1,** and **Fe2** and the doped **Cu3** and **Cu6** NPs. Low-intensity peaks (marked with an asterisk) in the **Cu3** and **Cu6** diffractograms closely resemble those of pristine **Cu** nanoparticles. (**right**) TEM images of the doped **Cu3** and **Cu6** NPs (see text for details).

Transmission Electron Microscopy (TEM) was used to investigate the morphology of the synthesized SCO NPs (Figure 1). The TEM images revealed that both samples, **Cu3** and **Cu6**, exhibit irregularly shaped elongated plates with an average dimension of 63 and 87 nm, respectively (Figure 1), and the Gaussian distribution of sizes is shown in Figure S2. Comparable dimensions were reported for the pristine [Fe(NH_2_trz)_3_]Br_2_ NPs reposted elsewhere [21,22]. IR spectroscopy (Figure S3, Table S3) and powder X-ray diffraction (p-XRD) (Figure 1) were employed for the structural characterization. The low crystallinity of the samples prevented reliable indexing. To check the doping scenario, a reverse micellar approach was followed to synthesize the coordination polymer [Cu(NH_2_trz)_3_]Br_2_ (**Cu**), and its p-XRD diffractogram is shown in the same figure for comparison reasons. The **Cu3** and **Cu6** samples display similar diffractograms. In the 10–18° 2θ region, low-intensity peaks (marked with an asterisk) closely resemble those of pristine **Cu** nanoparticles. Additionally, high-intensity peaks at 8°, 19.2°, and 22° correspond to the pristine iron nanoparticles (**Fe1** and **Fe2**). The high 2θ region of **Cu3** and **Cu6** features numerous overlapping peaks, preventing successful matching with **Cu**, **Fe1**, and **Fe1** diffractograms. The differences between the **Cu** and the pristine **Fe1, Fe2** diffractograms are due to the iron and copper complexes’ distinct crystal structures and coordination environments. Variations in bond lengths, angles, and overall crystal packing arise from the differences between metal ions (Fe and Cu), mainly due to the strong Jahn–Teller effect exhibited by Cu(II) ions. These characteristics are evident in their distinct p-XRD diffraction patterns. It should be noted here that similar differences have been reported for the p-XRD diffractograms of [Fe(NH_2_trz)_3_](NO_3_)_2_ and [Cu(NH_2_trz)_3_](NO_3_)_2_ CPs [23].

### 2.2. Magnetic Properties

**Figure 2:**
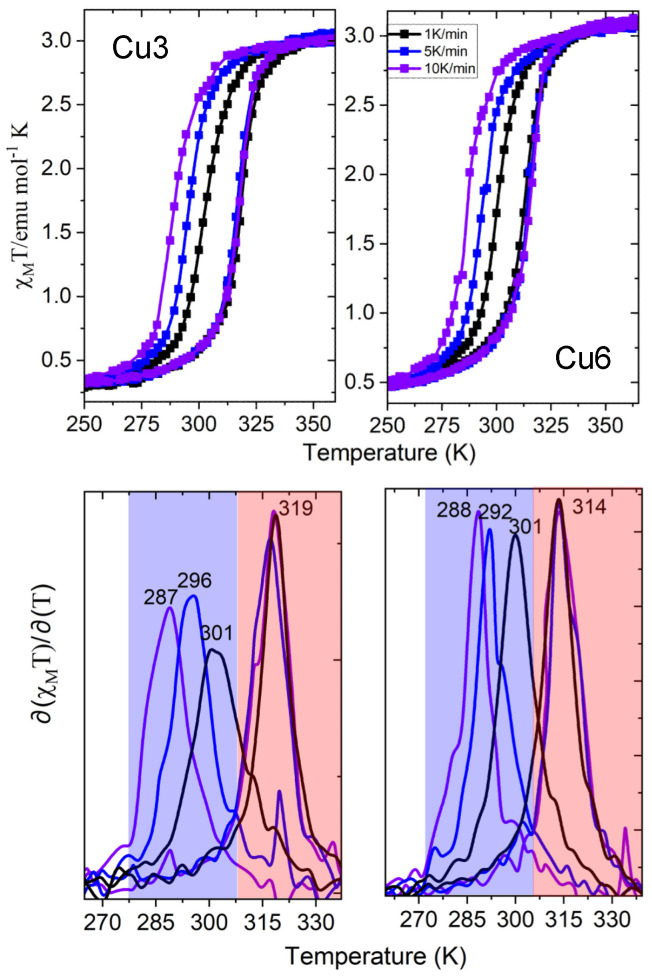
(**upper**) Temperature dependence of the susceptibility data, in the form of χ_Μ_Τ, of the dehydrated SCO NPs **Cu3** and **Cu6** (third cycle) for sweep rates of 1 K/min (black squares), 5 K/min (blue squares), and 10 K/min (violet squares). (**lower**) The first derivatives of thermal hysteresis are shown as solid lines. The derivatives of the heating branch are depicted in the orange boxes, while the violet boxes enclose the derivatives of the cooling branches.

The temperature dependence of the susceptibility data of **Cu3** and **Cu6**, in the form of χ_Μ_Τ, and at different scan rates (1 K/min, 5 K/min, and 10 K/min) is shown in Figure 2, along with the determination of the critical temperatures derived from the derivatives of the susceptibility curves. The room temperature value of χ_Μ_Τ is close to 3.1 emu mol^−1^ K for both **Cu3** and **Cu6**, confirming the population of the HS state, while at low temperatures, a small percentage of HS as well as **Cu** impurities (according to the pXRD studies) exists (5% and 10%, respectively). The experimental protocol followed is based on a pre-heating stage of the samples at 400 K to ensure their dehydrated nature and the performance of three or more thermal cycles in the magnetometer using the sequence 200 K–400 K–200 K to stabilize the repeatability of the SCO phenomenon. No steps appear in the thermal hysteresis of samples **Cu3** and **Cu6** in contrast to the non-doped pristine [Fe(NH_2_trz)_3_] NPs presented elsewhere [21]. Possibly, the doping scenario prevents the coexistence of different polymorphs or chains with various lengths, which has been proposed as a possible reason for the two-step hysteretic behavior of the non-doped pristine [Fe(NH_2_trz)_3_] NPs [24,25].

More explicitly, for the slow magnetic scan rate of 1 K/min, the critical temperatures are T_1/2_↑ = 319 K/314 K and T_1/2_↓ = 301 K/301 K, with hysteresis widths of 18 K/13 K for **Cu3**/**Cu6**, respectively. Increasing the scan rate to 5 K/min, the critical temperatures T_1/2_↑ remained unchanged while T_1/2_↓ = 296 K/292 K, increasing the hysteresis widths to 23 K/22 K, respectively. For the fastest scan rate of 10 K/min, the critical temperatures T_1/2_↑ remained once again unchanged while T_1/2_↓ = 287 K/288 K, with hysteresis widths of 32 K/26 K, respectively. It should be pointed out that for the case of pristine [Fe(NH_2_trz)_3_] Br_2_ NPs, two-step hysteretic behavior is noted at low magnetic sweep rates (1 K/min) for a reaction duration of 20 h (and ω_0_ = 4, 10), while a significant alteration in the magnetic properties is observed for a reaction time of 2 days and ω_0_ = 10 where the NPs maintain a one-step hysteresis across all sweep rates with an unshifted T_1/2_↑ = 335 K and a notable hysteresis-width of 45 K at 10 K/min. For comparison reasons, the susceptibility data of the pristine [Fe(NH_2_trz)_3_] Br_2_ NPs, **Cu3**, and **Cu6** at different magnetic sweep rates are presented in Figure S4.

The magnetic measurements revealed a sweep rate dependence of the hysteretic curve, according to which the cooling branch shifts to lower temperatures with the increase of the scan rate from 1 K/min to 10 K/min. At the same time, the heating component remains unshifted, and there is an overall increase in the coercivity width. Due to long metastable state lifetimes [26,27], the pristine [Fe(NH_2_trz)_3_] Br_2_ NPs have also observed this behavior. In conclusion, the Cu-doped samples **Cu3** and **Cu6** reveal an SCO phenomenon at lower critical temperatures, with narrowed hysteresis loops and a less abrupt spin transition than the pristine [Fe(NH_2_trz)_3_] NPs.

Differential scanning calorimetry (DSC) was performed to explore the spin transitions of **Cu3** and **Cu6** over the temperature range of 250 to 400 K at a heating rate of 10 K/min. The findings after the third thermalization cycle are depicted in Figure S5. Table S4 presents the calculated exothermic and endothermic peaks, along with the critical temperatures derived from the derivatives of the susceptibility curves of **Cu3** and **Cu6**, which are in close agreement.

### 2.3. EPR Study

**Figure 3:**
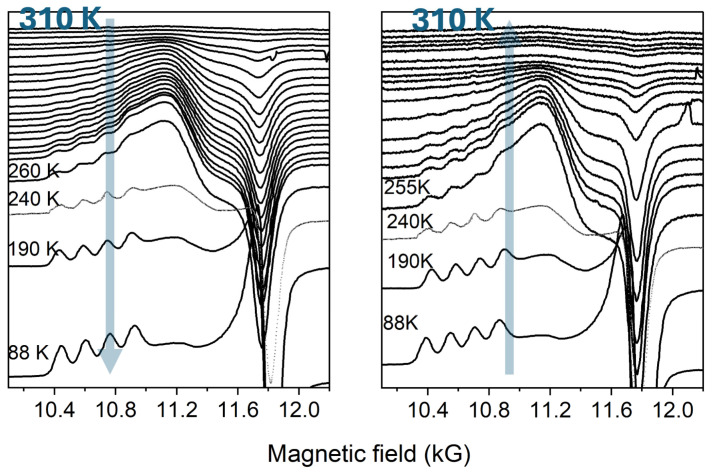
Temperature-dependent EPR spectra of the Cu-doped sample **Cu3** in both heating (88–310 K) and cooling fashion (310–88 K) (see text for details).

Temperature-dependent EPR measurements were carried out for the Cu-doped **Cu3** sample to monitor the SCO phenomenon. Because only small percentages (≤3%) of the dopant ion are required [6,16,17] to identify the spin transitions in EPR experiments, we decided to focus our study on the **Cu3** NPs. Representative spectra in heating (88–310 K) and cooling fashion (310–88 K) are shown in Figure 3. As shown in Figure 3, the Cu-doped sample’s low-temperature EPR spectrum (88 K) is typical of a tetragonally distorted octahedral environment due to the Jahn–Teller effect. It exhibits g anisotropy and a resolved hyperfine quadruplet in the parallel orientation of the applied magnetic field B. The usual spin Hamiltonian H = βS ·g·B + S ·A ·I applies, where g and A are, respectively, the g-tensor and the hyperfine structure tensor. No superhyperfine splitting from neighboring nitrogen ions can be discerned. As the temperature is raised towards that of the spin transition, the amplitude of the low-temperature spectrum diminishes without any apparent broadening or shift. On the contrary, a new asymmetric broadened signal, exhibiting no resolved hyperfine structure, grows at the expense of the low-temperature one. Above the spin transition temperature, where the Fe^2+^ is high spin, the broad EPR spectrum dominates and is further broadened and diminished due to dipolar interaction with Fe(II) (S = 2).

The recorded EPR spectra of all samples at various temperatures were analyzed best as comprising two components of two types of isolated atomic Cu(II) sites. Component **F** in which Cu(II) is found in a tetragonally distorted octahedral environment due to the Jahn–Teller (JT) effect with g parallel exhibiting the nuclear hyperfine splitting. Component **E** is where the Cu(II) signal appears significantly broadened with no discernible hyperfine splittings. It may originate from similar sites as in component **F** since their average <g> value is similar. Still, the signal shows larger linewidth, which may be attributed to dipolar broadening effects.

**Figure 4:**
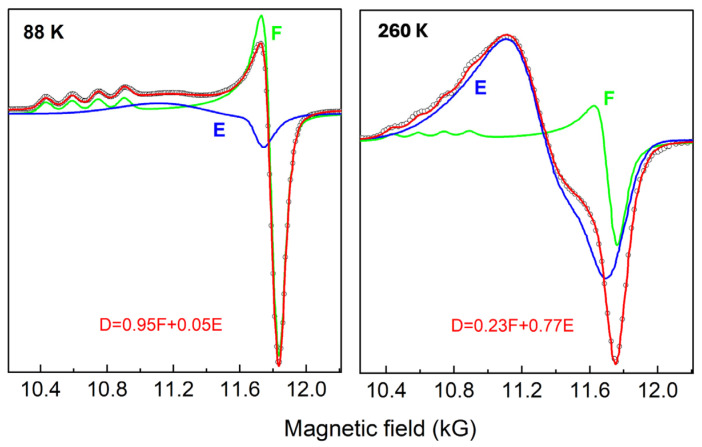
Spectral fitting (red line designated **D**) at two different temperatures, 88 and 260 K, in the heating mode, based on the composition of two components: (i) component **F** (green line) in which Cu(II) is in a tetragonally distorted octahedral environment with g parallel exhibiting the nuclear hyperfine splitting and (ii) component **E** (blue line) in which the Cu(II) signal appears significantly broadened with no discernible hyperfine splittings. The experimental spectra are presented as open cycles (see text for details).

**Figure 5:**
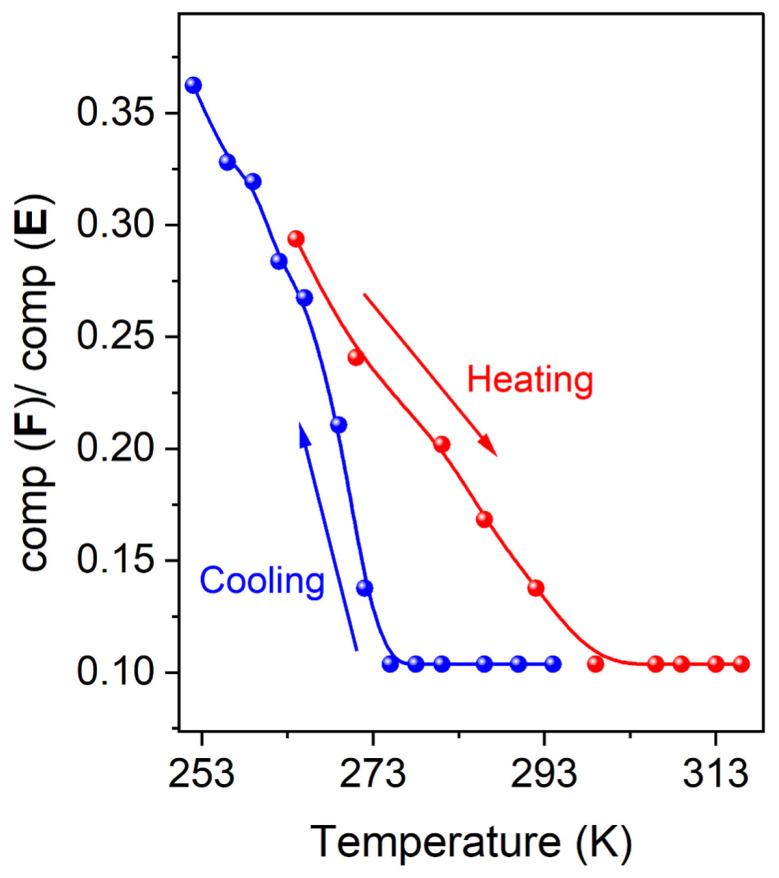
Temperature dependence of the ratio of the two components **E** and **F** in both heating and cooling fashion denoting the hysteretic behavior of the SCO phenomenon.

An example of spectral fitting is discussed below, while the simulation results in representative spectra at 88 and 260 K in the cooling mode are shown in Table 1 and Figure 4. The spin Hamiltonian parameters of the two components (**E**, **F**) at two different temperatures (88 K and 260 K in cooling mode) are presented in Table 1. It was found that the values of component **F** were almost stable for all the spectra in both the cooling and heating modes, while the values of component **E** (mostly g_1_) were temperature-dependent, indicating a temperature-dependent structural variation. The simulated spectra of the components **E** and **F** are presented in Figure 4, along with their combinations (depicted by spectra **D**) to simulate the experimental spectra. The EPR spectra of the Cu-doped sample, obtained at different temperatures in ascending or descending fashion, were analyzed as above. Each spectrum was best fitted using percentages of the components **E** and **F**, and the variation of the percentage’s ratio (**F**/**E**) was plotted against temperature (Figure 5), revealing the sensitivity of the EPR spectroscopy in monitoring the hysteretic nature of the SCO phenomenon.

Various models have been proposed to interpret the experimental results of the EPR spectra of Cu(II) ions in the [Fe(NH_2_trz)_3_]^2+^ compound and will be discussed in detail below.

**Jahn–Teller effect**: According to crystallographic reports [28,29], the average Fe-N distances in the [Fe(NH_2_trz)_3_]^2+^ compound are anticipated to range from 1.95 to 1.99 Å in the low-spin state and approximately 2.2 Å in the high-spin state. In contrast, for [Cu(NH_2_trz)_3_]^2+^, the Cu-N distances in the basal plane fall between 1.99 and 2.09 Å, while those in the axial positions are closer to 2.3–2.4 Å due to the Jahn–Teller effect. In the low-spin state at lower temperatures, the actual symmetry of the Cu(II) sites is reduced compared to the axial configuration, revealing low-symmetry distortions in the host sites. Consequently, the Cu ions experience static Jahn–Teller distortions, which result in noteworthy g-factor anisotropy and a well-resolved hyperfine quadruplet. It is important to note that a low-symmetry crystal field removes the degeneracy of the three valleys associated with the well-known “sun-warped Mexican hat” potential energy of the lowest vibronic ^2^E doublet, corresponding to three distinct orientations of the Jahn–Teller distortions [30,31]. At low temperatures, the Jahn–Teller complex is confined to the lowest valley. As temperature increases, the higher-energy valleys become populated, resulting in a phonon-induced rapid reorientation of the Jahn–Teller axis [30,31]. If the magnetic parameters in the various energy valleys are similar, this would lead to a motional averaging of the low-temperature anisotropic spectrum and a gradual transition from an anisotropic pattern to a motionally narrowed isotropic one at elevated temperatures. However, in our case, a coexistence of both patterns occurs.

**Energy distribution model:** Based on an energy distribution model of the Jahn–Teller effect [20] developed by O. Kahn, such a behavior can only be accounted for by assuming a continues distribution of Δ-values arising from disorder in the crystal due to imperfect crystallization of the polymeric [Fe(NH_2_trz)_3_]^2+^, where Δ is the characteristic energy difference between the lowest and the two higher energy valleys. Hence, at a given temperature, Cu(II) ions at sites with Δ ≤ kT will undergo transitions between energy valleys, resulting in a dynamically averaged EPR pattern, while those at sites with Δ > kT will not, exhibiting the low-temperature pattern.

**Interacting domains model:** Finally, a thermodynamic model is applied to interpret the spin transition in polycrystalline [Fe_x_Cu_1−x_(NH_2_trz)_3_](NO_3_)_2_ and [Fe_x_Cu_1−x_(Htrz)_2_(trz)](BF_4_) polymers and investigate the coexistence of anisotropic and isotropic EPR patterns at elevated temperatures [20]. According to this model, Fe^2+^ ions can exist in either a high-spin (S = 2) or low-spin (S = 0) state in the spin transition temperature range. Two limiting cases can describe the spatial arrangement of the two spin states. In the first case, Fe(II) ions found in the same spin state may form domains within the volume of the solid. In the second case, Fe(II) ions in either a high-spin or low-spin state may be randomly distributed within the volume of the solid. The dopant Cu(II) ions are expected to be distributed randomly and occupy iron sites. The recorded EPR spectrum will essentially be determined by the surroundings’ structure, which will depend on the spin state of Fe(II) sites. The majority will be high spin above the spin-state transition temperature and low spin below. Therefore, according to the first scenario of the spatial arrangement of iron sites, the Cu(II) ions in their vast majority will be found in either a high-spin domain of Fe(II) or a low-spin domain of Fe(II). On the contrary, only a small minority of Cu(II) ions will be found in the boundary between domains. The EPR spectrum of Cu(II) ions found in low-spin domains will be asymmetric axial with hyperfine structure (as mentioned above). In contrast, ions in high-spin domains will be broadened and featureless due to spin–spin interaction with the Fe(II) (S = 2) ions. The overall EPR spectrum will be a linear combination of an asymmetric with hyperfine structure and an isotropic pattern confirming that the spin transition occurs in domains populated by ions of the same (HS/LS) spin state.

### 2.4. Raman Study

**Figure 6:**
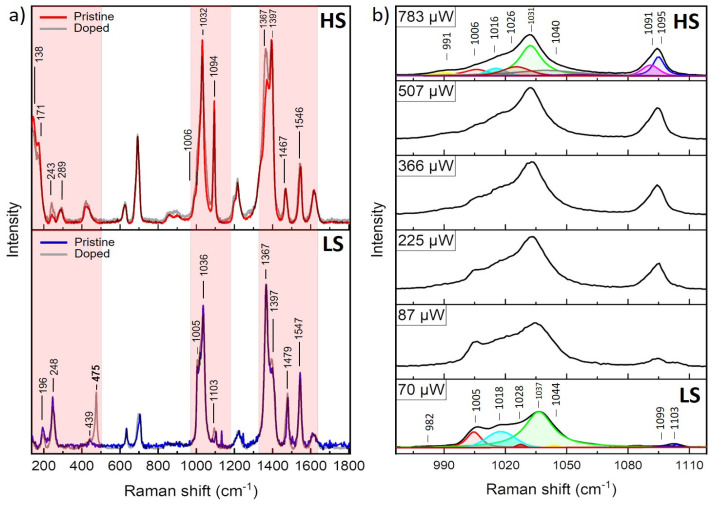
(**a**) Raman spectra for pristine **Fe** and doped **Cu3**, highlighting key spectral changes during the SCO transition, and (**b**) the evolution of Raman bands of the doped **Cu3** in the 950–1150 cm^−1^ region with laser power. At 70 μW (LS) and 783 μW (HS), the spectra include the characteristic Raman bands used for the fitting procedure (see text for details).

Raman spectroscopy has been suggested as an effective method for monitoring the spin-crossover (SCO) phenomenon in the Fe/triazole family of CPs [32,33,34,35,36,37]. Typically, temperature-dependent experimental protocols are employed to observe SCO behavior; however, there are only a few reports that concentrate on the use of laser power-assisted Raman spectroscopy. We have shown previously that laser-power-assisted Raman spectroscopy is sensitive to vibrational changes and is an effective tool for probing SCO transitions in Fe/NH_2_-triazole family of CPs [21,22]. Similarly, this study employed Raman microscopy to simultaneously trigger the SCO effect thermally by increasing the incident laser power and recording the resulting spectral changes. Typical **Fe** (**Fe1** or **Fe2**) and **Cu3** Raman spectra in the LS (derived at 70 mW) and HS (derived at 783 μW) are presented in Figure 6, with differences primarily in band intensities. **Cu3** sample displays intensity variations and a diminishing Raman band at 475 cm^−1^ under elevated laser powers, underscoring alterations in Fe–ligand interactions. These observations suggest that doping modulates the SCO transition by influencing ligand field dynamics. The vibrational regions 150–300 cm^−1^, 950–1150 cm^−1^, and 1300–1600 cm^−1^ in the LS/HS state will be described below based on DFT studies and polarized Raman scattering experiments performed on spin-crossover (SCO) polymers [Fe(NH_2_trz)_3_](X)_2_·nH_2_O where X = Cl, Br, BF_4_, and NO_3_ [29].

***The 150–300 cm^−1^ Region***: In the LS state, Raman peaks in the 150–300 cm^−1^ region primarily correspond to librations and translations of ligands involving CN-NH2 torsional vibrations coupled with ligand librations. In the HS state, these peaks exhibit a red shift of approximately 100 cm^−1^, attributed to weakened Fe–ligand interactions, rendering them undetectable within the spectrometer’s range (Figure 6a). These spectral changes serve as fingerprints of the LS to HS transition.

***The 950–1150 cm^−1^ Region:*** In LS, vibrations include N–N and C–NNH_2_ stretching and NCN, NNC, and CNC bending within the triazole (trz) ring. Specific modes correspond to combinations of N–N stretching and ring deformations. In the HS state, a pronounced blue shift is observed in some peaks, particularly N–N stretching vibrations. For instance, bands at 1006 and 1040 cm⁻¹ in the LS state shift by over 60 cm^−1^, forming new bands around 1090 cm^−1^. This shift signifies stronger N–N bonds in the HS state, attributed to reduced charge transfer between Fe and nitrogen atoms. As shown in Figure 6b, increasing laser power enhances the intensity of these peaks, making them a fingerprint for the LS to HS transition, which is used in this study to quantify the HS fraction as a function of the incident laser power.

***The 1300–1600 cm^−1^ Region:*** In the LS state, vibrations involve C–N, C–NH_2_, and C–NNH_2_ stretching, as well as H–C–N bending, while in the HS state, Raman bands broaden and shift to lower wavenumbers. The C–N stretching band at 1479 cm^−1^ undergoes a red shift (~13 cm^−1^), while the band at 1547 cm^−1^ (C–NNH_2_ stretching) remains largely unaffected. These changes serve as an additional fingerprint for the LS to HS transition (Figure 6a). Based on the above description, we summarize in Table S5 the prominent Raman bands and their assignments for the LS and HS states.

**Figure 7:**
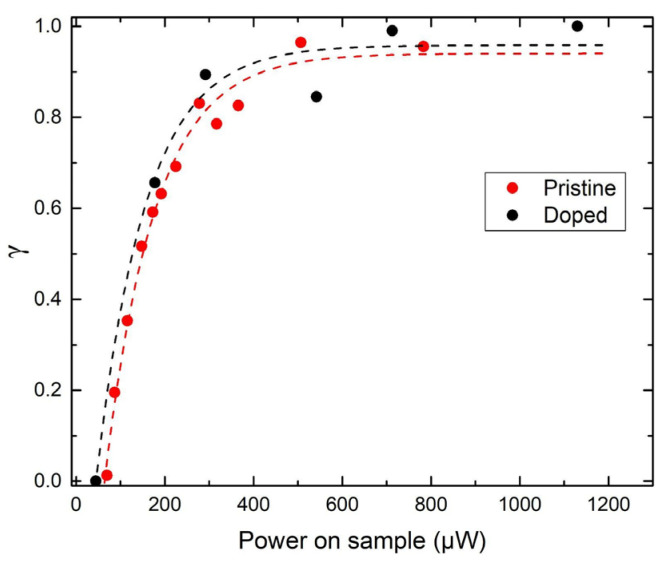
The dependence of the HS fraction on laser power for both pristine **Fe** and doped sample **Cu3** enables a quantitative understanding of the spin transition. The dashed curves are guides to the eye.

The spectral evolution in the region 950–1150 cm^−1^ for the pristine **Fe** and doped **Cu3** at elevated laser powers was carried out and presented in Figure 6 and Figure S6. The quantification of the HS fraction, $\gamma =\frac{\lambda -\lambda_{LS}}{\lambda_{HS}-\lambda_{LS}}$, was performed using the integrated intensities of characteristic Raman bands. Specifically, bands centered around ~1020 cm^−1^ and ~1100 cm^−1^ in the 950–1150 cm^−1^ region were selected for analysis (Figure 6b and Table S5). The Raman spectra were fitted using Voigt profiles to resolve these peaks accurately. The intensity ratio λ was calculated as the ratio of the integrated intensities of the Raman complex at 1020 cm^−1^ to that at 1100 cm^−1^. This ratio was then used to determine γ, representing the HS state’s relative population within the laser spot area (~1 μm diameter). Figure 6b illustrates the fitting procedure, highlighting the resolved peaks in both the LS and HS states. The calculated γ values were plotted as a function of laser power, as shown in Figure 7. This approach provides a quantitative measure of the SCO transition dynamics under varying excitation conditions. The integrated area ratio, γ, as a comparative metric, was found to mostly agree with the heating step of a magnetic susceptibility measurement hysteresis loop (Figure S4). At 700–800 μW laser power during heating, the doped sample **Cu3** and the pristine **Fe** reached % HS population of 100%. At the same time, the **Cu3** completed the HS conversion faster than the **Fe**, which is also evident in the thermal hysteresis loop (Figure S4).

## 3. Materials and Methods

### 3.1. Materials–Instrumentation–Physical Measurements

All experimental procedures were performed using commercial reagents and solvents at ambient aerobic conditions. The water was triple deionized (3DI) and deoxygenated after argon bubbling. The organic phase (11.1 mL) is a clear homogeneous solution with cyclohexane (7.5 mL), TX100 (1.8 mL), and 1-hexanol (1.8 mL).

IR spectra (4000–400 cm^−1^) were recorded using a (Perkin-Elmer 16PC FTIR, Waltham, MA, USA) spectrometer with samples prepared as KBr pellets.

EPR measurements at Q-band were performed on a home-assembled spectrometer with an ER 5106 QT Bruker resonator (Bruker, Karlsruhe, Germany), an Anritsu MF2414C microwave frequency counter (Anritsu, Atsugi, Japan), and a CF935P (Oxford Instruments, Abingdon, Oxfordshire, UK) helium cryostat. The temperature was controlled using an Oxford ITC 4 (Oxford Instruments, Abingdon, Oxfordshire, UK) temperature controller. A gold iron (0.03 atomic %) and chromel thermocouple were placed directly above the sample for exact temperature measurements. The sample was cooled using a slow current of nitrogen gas from a tank of liquid nitrogen and was warmed using the heater of the ITC temperature controller. A few milligrams of powders of each sample were loaded into quartz EPR tubes (3.0 mm o.d., 2.0 mm i.d.) for measurements. All EPR spectra were collected at variable temperatures over a wide magnetic field range. A typical experimental run would entail sample equilibration at the starting temperature (either high or low end) for at least 30 min, spectrum acquisition time of 3 min, and temperature change time of about 4 min. Typical spectrometer parameters were as follows: center field, 12,000 G; sweep width, 5000 G; microwave power, 0.4 mW; microwave frequency, 34 GHz; modulation frequency, 100 kHz. The EPR spectra were analyzed and simulated using MATLAB’s Easyspin program software v. 6.0 (https://easyspin.org, accessed on 30 January 2025) [38].

All Raman measurements were conducted under ambient conditions in a moderately humid environment without using inert or additional gases like nitrogen. Raman measurements were performed using a Renishaw inVia Raman spectrometer (Renishaw, Wotton-under-Edge, Gloucestershire, UK) in the backscattering geometry. A solid-state 515 nm laser (Cobolt Fandango 50) (Hubner Photonics, Kassel, Germany) was focused using a LEICA 50× objective lens (NA 0.75) (Leica, Wetzlar, Germany) producing a laser spot size of approximately 1 μm. Laser power on the sample was adjusted using a variable neutral density optical filter (mks|Newport 25FS04DV.4) and monitored with a Newport 1918-R power meter (Newport Corporation, Irvine, CA, USA). Raman scattered radiation was dispersed using a 2400 grooves/mm grating, and an edge filter allowed the detection of Raman shifts greater than 100 cm^−1^. The Raman microscope facilitated simultaneous thermal triggering of the SCO effect and spectral recording.

**Laser-power-dependent methodology:** In our laser power dependence study, we conducted a controlled heating cycle ranging from 70 μW (~0% HS) to 783 μW (~100% HS). After reaching a 100% high-spin (HS) population (γ ~ 1) at 783 μW, we immediately measured 51 μW. Despite this significant reduction in laser power, the Raman spectral profile remained indicative of the HS state, corresponding to an HS fraction γ = 0.796. This result is reasonable when considering the hysteresis of the SCO transition, where cooling occurs below ambient temperatures. Even at very low laser powers, the local heating effect maintains a high HS fraction. Once the sample reaches 100% HS conversion, active cooling is required to revert to 0% HS, regardless of the applied laser power. This behavior is governed by the thermal stability of the HS state, which does not spontaneously revert under ambient conditions unless adequate cooling is introduced. We allowed the sample to cool naturally for 18 h under ambient conditions to verify this behavior. A subsequent Raman measurement at 70 μW on the same sample area confirmed the complete reversibility of the transition as a 100% LS spectral profile was once again observed.

These results demonstrate that while immediate reversibility through laser power modulation alone may not be possible due to local heating effects, the SCO transition is fully reversible when the sample is given sufficient time to equilibrate thermally. This behavior aligns well with the inherent thermal hysteresis of the SCO systems under investigation, showcasing the stability and reliability of our methodology in exploring the photo-induced SCO transition.

The powder X-ray diffraction (PXRD) measurements were performed at room temperature on a Bruker D8 Advance diffractometer (Bruker, Karlsruhe, Germany) with focusing Kα1 geometry. Polycrystalline samples were loaded in 1 mm borosilicate glass capillaries while the X-ray tube operated at 45 kV and 40 mA. The incident-beam side (CuKα1 radiation, λ = 1.54056 Å) has a focusing X-ray mirror.

The TEM study was performed utilizing an FEI CM20 TEM (Thermo Fisher Scientific, Waltham, MA, USA) operating at 200 kV. TEM specimens were prepared by drop-casting a 3 μL droplet of nanoparticle suspension in acetone on a carbon-coated Cu TEM grid. The size of the particles is determined by “manual counting” using ImageJ software, v. 1.54d (https://imagej.net, accessed on 10 December 2024).

The direct-current (DC) magnetic susceptibility measurements were measured on powder samples using a physical-properties measurement system (PPMS, Quantum Design, San Diego, CA, USA) at 200–400–200 K thermal loops with a rate of 1.0/5.0/10.0 K min^−1^ under an applied dc magnetic field of 1000 Oe. The experimental data were corrected for the diamagnetism and signal of the sample holder, and the Pascal constants were used for the diamagnetic corrections.

Differential scanning calorimetry (DSC) measurements were carried out in an N-(g) atmosphere using a DSC (Q100, TA Instruments, New Castle, DE, USA) instrument. Aluminum hermetic pans encapsulated 5–7 mg of the sample. The pans were purged with nitrogen at a rate of 50 mL·min^−1^, and liquid nitrogen was used for cooling. Initially, the samples were cooled down from 253 K to 410 K at 10 K/min. Then, the samples were subjected to three successive thermal regimes of (a) heating from 253 K to 410 K at 10 K min^−1^ and (b) cooling to 253 K at the same rate.

### 3.2. Preparation of Nanoparticles **Cu**, **Cu3**, and **Cu6**

**[Cu(NH_2_trz)_3_](Br)_2_^.^3H_2_O^.^0.02TX100 (Cu):** An aqueous solution (0.5 mL 3DI H_2_O) of CuBr_2_ (223 mg, 1.0 mmol) is added slowly to the organic phase (11.1 mL). The resulting biphasic solution is stirred vigorously at room temperature until the microemulsion (A) is formed. Similarly, the microemulsion (B) is formed after mixing an aqueous solution (0.5 mL 3DI H_2_O) of NH_2_trz (252 mg, 3.0 mmol) with the organic phase (11.1 mL). After mixing microemulsions (A) and (B), the final blue microemulsion was left under magnetic stirring for 24 h. The reaction was completed with the addition of acetone. The precipitant NPs have an intense blue color. The product was washed thoroughly with EtOH (4 × 10 mL) and Me_2_CO (1 × 10 mL) and dried in air. Yield 300 mg. Elemental analysis: C[%] 14.61; H[%] 3.38; N[%] 31.12; assigned to molecular formula [Cu(NH_2_trz)_3_]Br_2_^.^3H_2_O^.^ 0.02TX100, Mr = 542.1 g/mol. Calc. values, C: 14.71%; H: 3.58%; and N: 31.01%.

**Fe_0_._97_Cu_0_._03_(NH_2_trz)_3_]Br_2_^.^2H_2_O^.^0.02TX100 (Cu3)**: An aqueous solution (0.5 mL 3DI H_2_O) containing FeBr_2_ (209.5 mg, 0.97 mmol), CuBr_2_ (6.7 mg, 0.03 mmol), and a small amount of ascorbic acid (5 mg) is added slowly to the organic phase (11.1 mL). After vigorous stirring, the microemulsion (A) is formed. Similarly, microemulsion (B) formed after mixing an aqueous solution (0.5 mL 3DI H_2_O) of NH_2_trz (252 mg, 3.0 mmol) with the organic phase (11.1 mL). The final microemulsion formed after mixing microemulsions (A) and (B) and left under magnetic stirring for 24 h. The reaction was completed with the addition of acetone. The precipitant NPs have a pink color. The product was washed thoroughly with EtOH (4 × 10 mL) and Me_2_CO (1 × 10 mL) and dried in air. Yield 290 mg. Elemental analysis: C[%] 15.63; H[%] 3.18; N[%] 32.22; assigned to molecular formula [Cu(NH_2_trz)_3_]Br_2_^.^2H_2_O^.^0.02TX100, Mr = 516.3 g/mol. Calc. values, C: 15.49%; H: 3.36%; and N: 32.55%.

**[Fe_0_._94_Cu_0_._06_(NH_2_trz)_3_]Br_2_^.^2H_2_O^.^0.02TX100 (Cu6)**: An aqueous solution (0.5 mL 3DI H_2_O) containing FeBr_2_ (203.0 mg, 0.94 mmol), CuBr_2_ (13.4 mg, 0.06 mmol), and a small amount of ascorbic acid (5 mg) is added slowly to the organic phase (11.1 mL). After vigorous stirring, the microemulsion (A) is formed. Similarly, microemulsion (B) is formed after mixing an aqueous solution (0.5 mL 3DI H_2_O) of NH_2_trz (252 mg, 3.0 mmol) with the organic phase (11.1 mL). After mixing microemulsions (A) and (B), the final microemulsion is formed and left under magnetic stirring for 24 h. The reaction was completed with the addition of acetone. The precipitant NPs have a dark-pink color. The product was washed thoroughly with EtOH (4 × 10 mL) and Me_2_CO (1 × 10 mL) and dried in air. Yield 300 mg. Elemental analysis: C[%] 15.71; H[%] 3.54; N[%] 32.27; assigned to molecular formula [Cu(NH_2_trz)_3_]Br_2_^.^2H_2_O^.^0.02TX100, Mr = 516.3 g/mol. Calc. values, C: 15.48%; H: 3.35% and N: 32.52%.

## 4. Conclusions

The study focused on the Fe(II) spin transition in Cu(II)-doped 1-D spin-crossover (SCO) nanoparticles of the type [Fe_1−x_Cu_x_(NH_2_trz)_3_]Br_2_, synthesized using a reverse micellar approach. Magnetic susceptibility measurements showed that the Cu(II) dopants influenced the thermal hysteresis behavior, with critical temperatures and widths varying with the scan rate. Specifically, the Cu-doped samples exhibited lower critical temperatures and narrower hysteresis loops than pristine Fe(II) nanoparticles. The study also demonstrated that the Cu(II) doping scenario prevents the coexistence of different polymorphs or chains with various lengths, which was proposed as a reason for the two-step hysteretic behavior observed in non-doped pristine [Fe(NH_2_trz)_3_] NPs.

EPR spectroscopy provided insights into the structural changes and spin-state transitions, revealing that the spin transition occurs in domains populated by ions of the same spin state. The Cu(II) ions displayed different spectral characteristics depending on whether they were in high-spin or low-spin domains of Fe(II). The EPR spectra analysis indicated that the Cu^2+^ ions in low-spin domains exhibited an asymmetric axial spectrum with a hyperfine structure. In contrast, those in high-spin domains showed broadened, featureless spectra due to spin–spin interactions with Fe^2+^ ions. 

Raman spectroscopy reveals vibrational fingerprints of the LS to HS transition, while key spectral changes in the 150–300 cm^−1^, 950–1150 cm^−1^, and 1300–1600 cm^−1^ regions reflect metal–ligand dynamics and electronic reconfigurations. Quantifying the HS fraction (γ) highlights the potential of Raman spectroscopy as a tool for characterizing SCO materials and their transitions.

Overall, the study provided valuable insights into the structural and magnetic properties of Cu(II)-doped SCO nanoparticles, enhancing the understanding of spin-crossover phenomena and the role of dopants in these materials.

## Figures and Tables

**Table 1:** Simulation results of spectral fitting at two temperatures, 88 and 260 K (heating mode).

Temperature (K)	Component E ^1^	Component F ^2^	Composition
**88**	(2.057, 2.139, 2.175) (1, 1, 210)	(2.272, 2.069) (465, 16)	95% **F**, 5% **E**
**260**	(2.076, 2.153, 2.211) (1, 1, 210)	(2.272, 2.069) (465, 16)	23% **F**, 77% **E**

^1^ Component **E** is presented as (g_1_, g_2_, g_3_) and (A_1_, A_2_, A_3_), where g and A values (MHz) are rhombic. ^2^ Component **F** is presented as (g_//_, g_⊥_) and (A_//_, A_⊥_), where g and A values (MHz) are axial.

## Data Availability

Data are contained within the article and Supplementary Materials.

## Supplementary Materials

The following supporting information can be downloaded at https://www.mdpi.com/article/10.3390/molecules30061258/s1, Table S1: Elemental analyses for **Cu3** and **Cu6**, the table depicts the ion contents for all the measurements; Figure S1: Iron(II) and Cu(II) ion content for the **Cu3** and **Cu6** determined by energy-dispersive spectroscope (EDS) of field-emission scanning electron microscope (3rd time); Table S2: EDS analysis for **Cu3** and **Cu6**; Figure S2: Gaussian distributions of sizes for the **Cu3** and **Cu6** NPs; Figure S3: IR spectra of the **Cu3**, **Cu6**, pristine **Cu,** and pristine **Fe** NPs; Table S3: Assignment of the observed bands in the IR spectra of the **Cu3**, **Cu6**, **Cu**, and **Fe**; Figure S4: Thermal loops (3rd cycle) at different magnetic sweep rates 1 K/min, 5 K/min, and 10 K/min for **Fe1** (red solid spheres), **Fe2** (violet solid spheres), **Cu1** (black solid spheres), and **Cu2** (blue solid spheres) (see text for details); Figure S5: DSC curves of **Cu3**–**Cu6** presenting the 3rd heating-cooling cycle at 10 K/min; Table S4: General synthetic protocol, critical temperatures derived from the DSC measurements and from the thermal magnetic hysteresis for **Cu3** and **Cu6**; Table S5: Summary of Raman Bands in the 950–1150 cm^−1^ region; Figure S6: The evolution of Raman bands of the pristine **Fe** NPs in the 950–1150 cm^−1^ region with laser power. At 45 μW (LS) and 1130 μW (HS), the spectra include the characteristic Raman bands used for the fitting procedure.
