# Supplementary material for: Monitoring of Fe(II) Spin Transition in Cu(II)-Doped Spin-Crossover Nanoparticles
Source: Molecules. 2025 Mar 11;30(6):1258. doi: 10.3390/molecules30061258 (PMC11946675; doi:10.3390/molecules30061258)
Supplement: Supplementary file 1 [file molecules-30-01258-s001.zip › molecules-3478788-supplementary.pdf]

SUPPORTING INFORMATION

Article

Monitoring of Fe(II) Spin Transition in Cu(II)-Doped Spin-Crossover Nanoparticles

Alexander Charitos [1], Vassilis Tangoulis [1,*], John Parthenios [2], Ondrej Malina [3], Radim Mach [3], Nikolaos Ioannidis [4] and Nikolia Lalioti [1,*]

[1] Laboratory of Inorganic Chemistry, Department of Chemistry, University of Patras, 26504 Patras, Greece; charitos.al97@gmail.com

[2] Institute of Chemical Engineering Sciences (ICE-HT), Foundation for Research and Technology-Hellas (FORTH), 26504 Patras, Greece; jparthen@iceht.forth.gr

[3] Regional Centre of Advanced Technologies and Materials, Czech Advanced Technology and Research Institute (CATRIN), Palacký University Olomouc, Šlechtitelů 27, 78371 Olomouc, Czech Republic; ondrej.malina@upol.cz (O.M.); radim.mach@upol.cz (R.M.)

[4] Institute of Nanoscience and Nanotechnology, NCSR "Demokritos", 15310 Athens, Greece; n.ioannidis@inn.demokritos.gr

* Correspondence: vtango@upatras.gr (V.T.); lali@upatras.gr (N.L.)

Elemental Analysis

Table S1. Elemental analyses for **Cu3** and **Cu6**

Sample NP		C [%]	N [%]	H [%]	Molecular Formulae
**Cu3**	exptl	**15.63**	**32.22**	**3.18**	$\mathbf{[Fe_{0.97}Cu_{0.03}(NH_2trz)_3]Br_2{\cdot}2H_2O{\cdot}0.02TX100}$
	calcd	15.49	32.55	3.36	516.66 g/mol
**Cu6**	Exptl	**15.71**	**32.27**	**3.54**	$\mathbf{[Fe_{0.94}Cu_{0.06}(NH_2trz)_3]Br_2{\cdot}2H_2O{\cdot}0.02TX100}$
	Calcd	15.48	32.52	3.35	516.88 g/mol

EDS Analysis

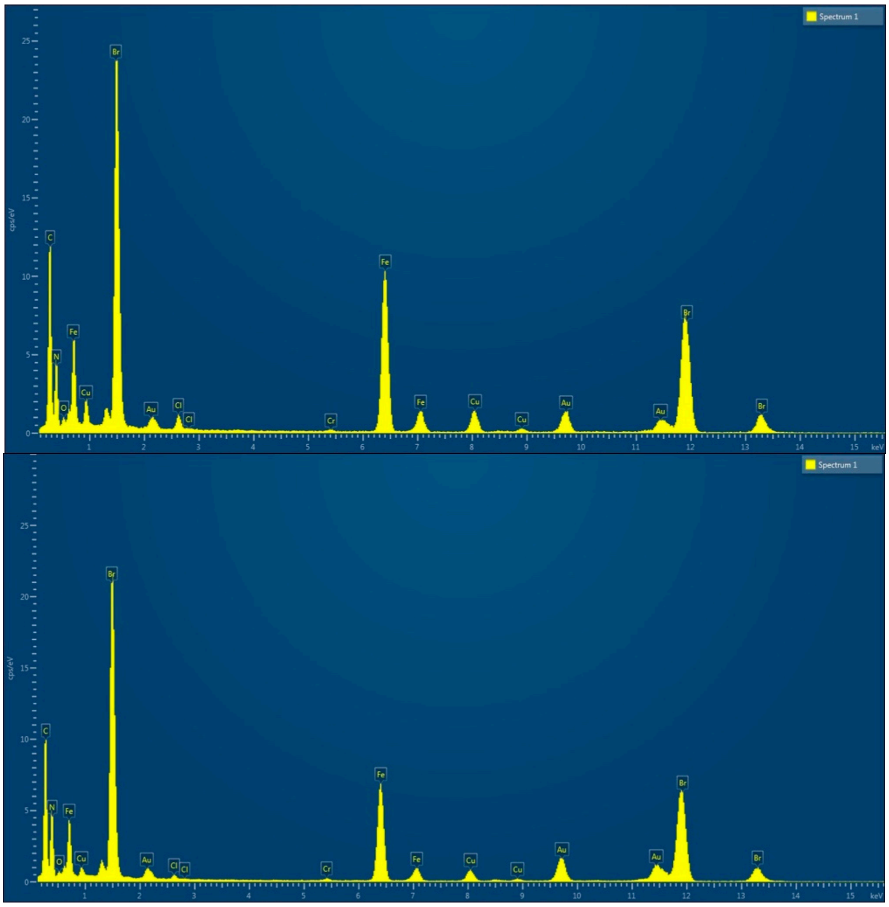

Figure S1. Iron(II) and Cu(II) ion content for the **Cu3** and **Cu6** determined by energy dispersive spectroscope (EDS) of field-emission scanning electron microscope (3rd time). Table S2 depicts the ion contents for all the measurements.

Table S2. EDS analysis for **Cu3** and **Cu6**

NPs	element	First Time %	Second Time %	Third Time %
**Cu6**	**Fe**	94.33	93.20	94.52
	**Cu**	5.77	6.80	5.48
**Cu3**	**Fe**	97.24	96.33	97.44
	**Cu**	2.76	3.67	2.56

TEM Study

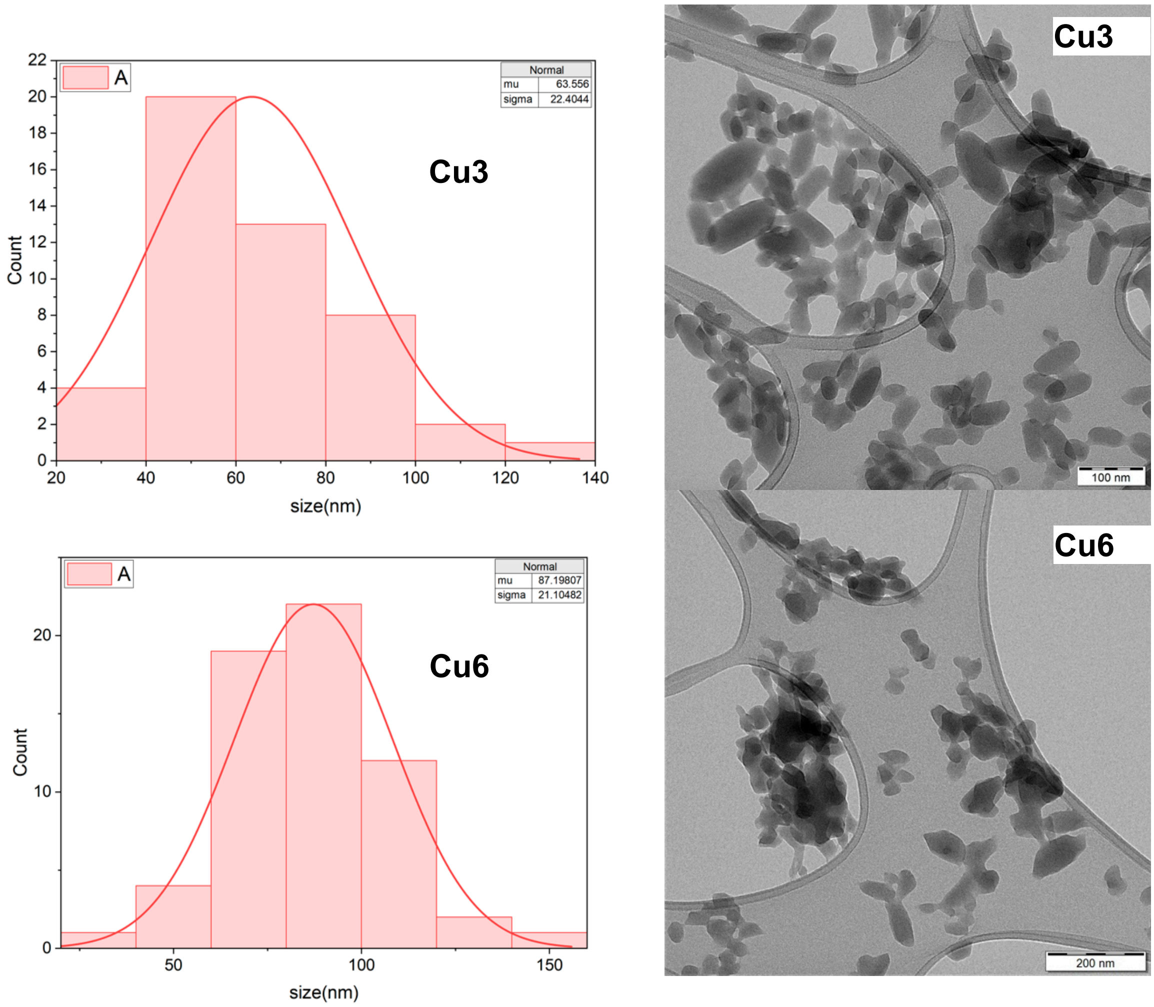

Figure S2. Gaussian distributions of sizes for the **Cu3** and **Cu6** NPs

IR Spectra

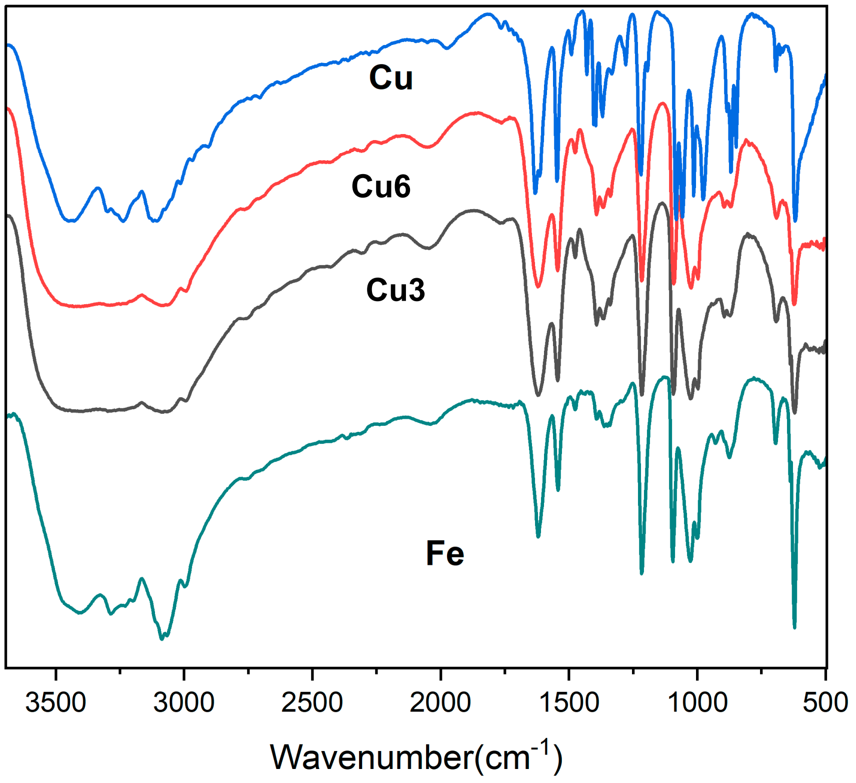

Figure S3. IR spectra of the **Cu3, Cu6**, pristine **Cu** and pristine **Fe** NPs**.**

Table S3. Assignment of the observed bands in the IR spectra of the **Cu3, Cu6, Cu, Fe.**

**[Fe(atrz)$_3$](Br)$_2$**	**description,**	**Fe(atrz)$_3$](Br)$_2$**	**description,**
3410s-3285 s, br	Vas(NH$_2$) Vs(NH$_2$) v(OH)water	1093s	δ(CH)
3085s	V(CH) stretch	1026s	R_6 ring stretch
1620s	δ(NH$_2$)	1000m	R_7 ring breathing
1545s	R_1 ring stretch	891m, br	γ(CH) bend
1475m	R_2 ring stretch	876m	β(NH$_2$)
1390m, br	R_3 ring stretch	693m	R_8 ring torsion
1215s	V(N-NH$_2$)	621s	R_9 ring torsion

m = medium, w = weak, br = broad, and s = strong

Magnetic Measurements

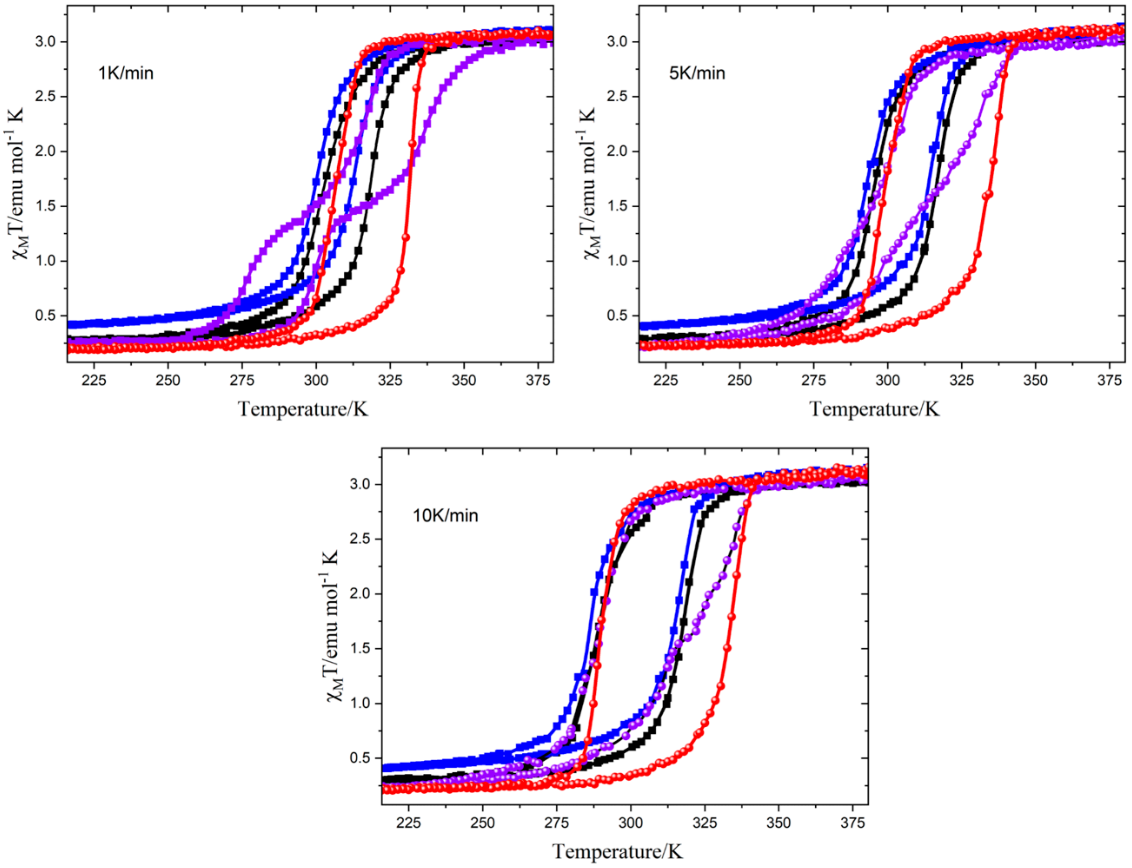

Figure S4. Thermal loops (3rd cycle) at different magnetic sweep rates 1K/min, 5 K/min and 10 K/min for **Fe1** (red solid spheres), **Fe2** (violet solid spheres), **Cu1** (black solid spheres) and **Cu2** (blue solid spheres). (see text for details).

DSC Measurements

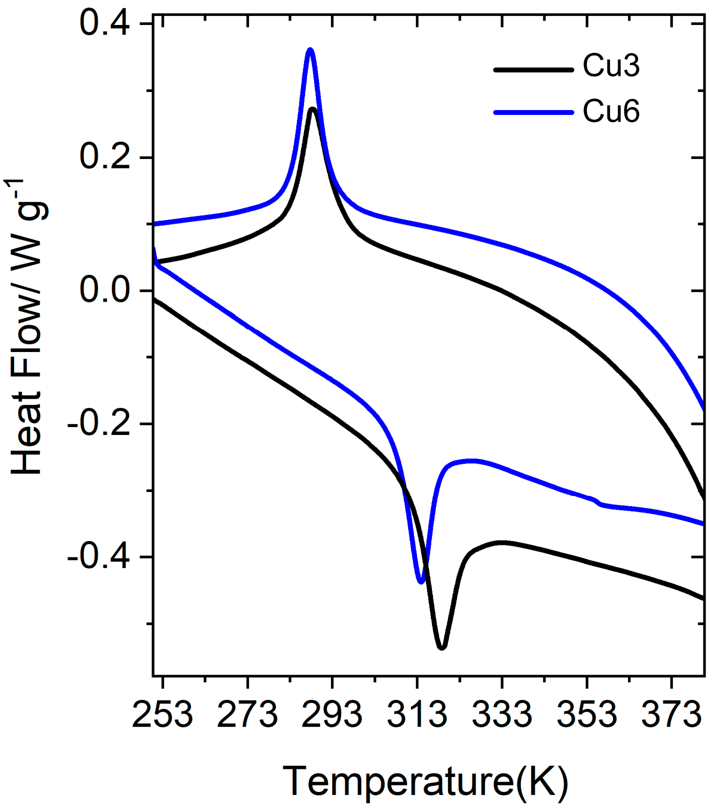

Figure S5. DSC curves of **Cu3-Cu6** presenting the 3rd heating-cooling cycle at 10 K/min.

Table S4. General synthetic protocol, critical temperatures derived from the DSC measurements and from the thermal magnetic hysteresis for **Cu3** and **Cu6**.

**NPs**	**ω**	**t**	**DSC**			**MAGNETIC**		
			**T_{up} (K)**	**T_{down} (K)**	**ΔT (K)**	**T_{up} (K)**	**T_{down} (K)**	**ΔT (K)**
							**1 K/min**	
**Cu3**	10	20 h				319	301	18
							**5 K/min**	
						319	296	23
				**10 K/min**			**10 K/min**	
			320	288	32	319	287	32
							**1 K/min**	
**Cu6**	10	20 h				314	301	13
							**5 K/min**	
						314	292	22
				**10 K/min**			**10 K/min**	
			313	286	27	314	288	26

Table S5. Summary of Raman Bands in the 950–1150 cm^{-1} region

Band Position (cm^{-1})	Spin State	Assignment
1005	LS, HS	N–N stretching of trz ring
1018	LS	N–N stretching and trz ring deformations
1037	LS, HS	trz ring deformations
1044	LS, HS	N–N stretching and trz ring deformations
1091	HS	Blue-shifted N–N stretching
1099	HS	Blue-shifted N–N stretching and ring deformations
1108	LS	N–N stretching and ring deformations

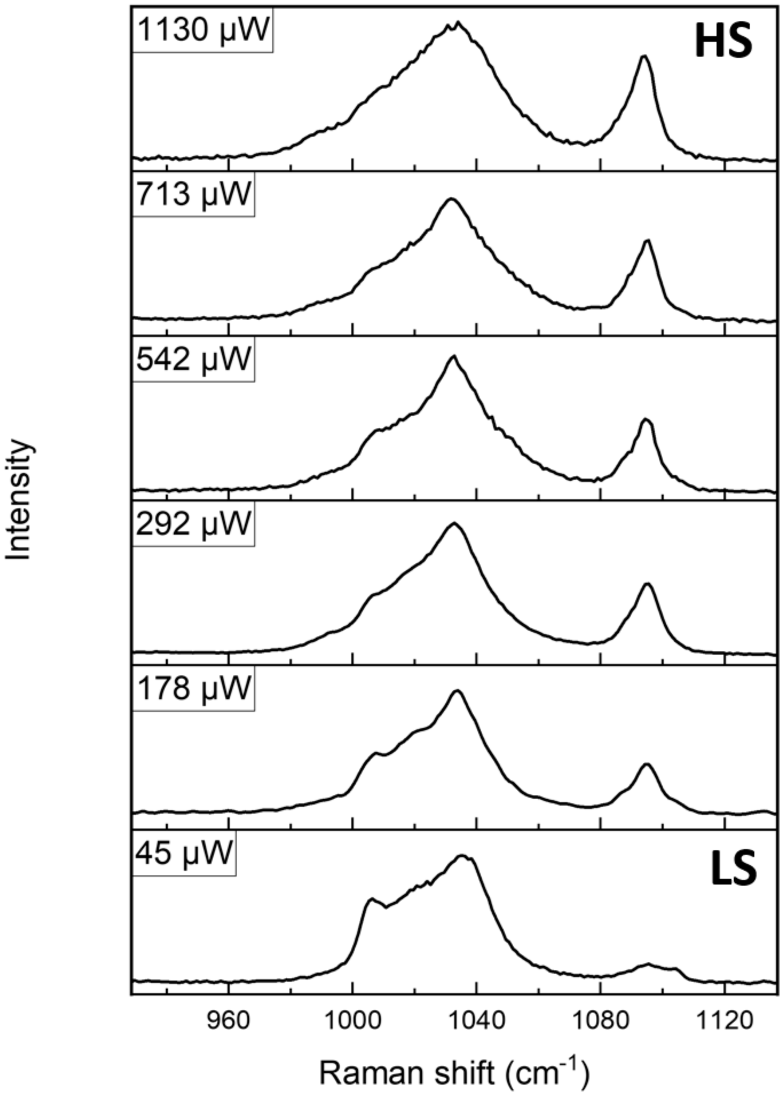

Figure S6. The evolution of Raman bands of the pristine **Fe** NPs in the 950–1150 cm^{-1} region with laser power. At 45 μW (LS) and 1130 μW (HS) the spectra include the characteristic Raman bands used for the fitting procedure.
